# Natural radioactivity and heavy metal contamination in edible fish, shellfish and mollusks at the Bay of Bengal, Kuakata, Bangladesh

**DOI:** 10.1016/j.heliyon.2024.e37787

**Published:** 2024-09-11

**Authors:** Samin Yeasar Risal, Saiful Islam, Jannatul Ferdous, Md Nure Alam Siddik, Pradip K. Bakshi

**Affiliations:** a Department of Chemistry, University of Dhaka, Dhaka 1000, Bangladesh; b Health physics Division, Atomic Energy Centre, Dhaka 1000, Bangladesh; c Bangladesh Council of Scientific and Industrial Research (BCSIR), Chattogram 4220, Bangladesh

**Keywords:** Natural radioactivity, Heavy metal, Edible fish, Shellfish, Mollusks, Annual effective dose, Bay of Bengal

## Abstract

In this study, gamma activity concentrations, gross alpha and gross beta activity of natural radionuclides, and heavy metal concentrations were measured in eleven edible marine fish, four shellfish, two mollusks, and a common seaweed sample collected from a local sea fish market and sea beach area of the northern part of the Bay of Bengal, Kuakata, Bangladesh. Using HPGe gamma spectrometry, the activity concentrations of ^238^U, ^232^Th, and ^40^K were measured and found to be 19.7 ± 1.5 Bq/kg, 12.2 ± 0.9 Bq/kg, and 188 ± 15 Bq/kg, respectively, with the ^232^Th concentration surpassing that of ^238^U. The ZnS scintillation detector was used to assess the gross alpha and gross beta activity. The average gross alpha activity and gross beta activity were found to be 9.4 ± 1.4 Bq/kg and 26 ± 4 Bq/kg, respectively, with the latter attributed to beta emitting radionuclides's abundance. The activity concentrations of ^238^U, ^232^Th, and ^40^K in the analyzed samples varied in the order of Shellfish > Seaweed > Fish > Mollusks, Seaweed > Shellfish > Mollusks > Fish, and Seaweed > Shellfish > Fish > Mollusks. The annual effective dose due to consumption of analyzed seafood was found to be within the world limit of 2400 μSv/y recommended by UNSCEAR. The main contributor to the annual effective dose was ^238^U. The excess lifetime cancer risk (ELCR) results were below the permissible threshold of 10^−3^ for radiological risks. Furthermore, average concentrations of Zn and Mn were higher than WHO/FAO recommended values, and carcinogenic Pb, Cd, Cr, and Hg concentrations were below detection limits, according to heavy metal analysis performed by AAS. The average concentration of heavy metals in all of the seafood samples under investigation was in the order of Zn > Fe > Mn. The calculated hazard index (HI), target hazard quotient (THQ), and estimated daily intake (EDI) were compared to the permissible safety limits.

## 1 Introduction

Radioactivity in the marine environment is a complex problem that has significant effects on human health as well as marine ecosystems. Due to natural processes and human activity, radioactivity has grown to be a major global concern in marine environments. All terrestrial minerals contain naturally occurring radioactive materials of ^226^Ra, ^232^Th, and their decay products, as well as a single radionuclide of ^40^K. These materials are widely distributed throughout the Earth's crust [1,2]. Ocean radioactivity has increased as a result of the release of radioactive isotopes, which can occur from routine nuclear facility discharges, accidents like Fukushima and Chernobyl, or nuclear weapons testing. To lessen the effects of radioactivity on marine ecosystems and human populations, it is essential to comprehend the routes of introduction and the effects on marine organisms. There are several ways that radioactivity can enter marine environments: upwelling currents that carry radioactive material from the ocean floor to the surface, runoff from terrestrial sources carrying radioactive contaminants, direct deposition from atmospheric fallout and emissions, and groundwater discharge from nuclear waste sites [3]. Furthermore, bioaccumulation is a process by which marine organisms take in and concentrate radioactive material from their environment and contributes significantly and causes biomagnification higher up the food chain [4]. Radiation exposure and trace metal contamination can harm marine life in various ways, including reduced reproductive success, morphological changes, genetic damage, and cell death. These consequences may upset the balance of the ecosystem, resulting in population decreases and a loss of biodiversity [2,5]. The direct danger that radioactive material contamination of seafood poses to human health emphasizes the relationship between radioactivity in the marine environment and human health [6]. Although heavy metals have always been present in seawater and sediments, the release of heavy metal-containing industrial waste into the marine environment may be the cause of the high concentration of heavy metals seen in some marine animals, including fish. Additionally, heavy metals can build up in marine life by direct absorption or through their food chain, which can then transfer to humans and cause disorders [7,8].Marine organisms are exposed to heavy metals including mercury (Hg), lead (Pb), cadmium (Cd), and arsenic (As) through a variety of processes such as atmospheric deposition, industrial runoff, and natural geological processes [9]. Heavy metals like Pb, Cd, and Hg have been found to bioaccumulate in marine life, especially in the lipid-rich tissues of organisms [10,11]. This poses a serious risk of contamination to the seafood chain. Similarly, it has been discovered that radioactive isotopes like ^90^Sr and ^137^Cs concentrate in the skeletal systems and muscles of fish, endangering both human and marine health [12].

In the previous studies, assessment of radioactivity level and contamination level of trace metals in sea fish collected from Chittagong and Cox's Bazar, the sea costal area of Bangladesh was carried out. In 2017, M.H. Kabir measured the radioactivity of ^238^U, ^232^Th, and ^40^K in sea fish, coral collected from St. Martin's Island, Chittagong, Bangladesh [13]. A. D. Gupta et al. carried out an experiment to assess the radiological exposure due to the intake of fishes and seafoods from some common estuary of Karnaphuli River and Bay of Bengal, Bangladesh in 2018 and found that the radioactivity was below the world average values [14]. In 2021, Biswas et al. measured the radioactivity concentration of ^226^Ra, ^232^Th, ^40^K and heavy metal concentration of Cd, Pb, Zn, Cu, Ni, Fe, Mn, and Cr in marine edible fish and crustaceans at the Bay of Bengal, Chattogram, Bangladesh [15]. They found that the radioactivity was below the world average values and heavy metals were observed in excessive contents in some of the analyzed samples. In the Bay of Bengal region, there is not much research on the natural radioactivity levels and heavy metal concentrations in the most common edible fish, shellfish, and mollusk species. There hasn't been any research done on the radioactivity and heavy metal contamination of seafood in the Kuakata area of the northern Bay of Bengal. The present study aimed to ascertain the level of radioactivity and heavy metal contamination in eleven marine edible fish, four shellfish, and two mollusk species found in the Kuakata area in the northern part of the Bay of Bengal, considering the significance of such knowledge. Common seaweed was also studied to understand more about the bioaccumulation of heavy metals and radionuclides on Kuakata's seashore. By using gamma spectrometry and atomic absorption spectrometry, respectively, the bioaccumulation level of natural radionuclides ^238^U, ^232^Th, and ^40^K as well as heavy metal contamination was evaluated. To evaluate the marine environmental quality in this area, a number of radiological hazard and heavy metals health-hazard metrics were computed in addition to the health concerns associated with consuming these contaminants.

## 2 Materials and methods

### 2.1 Sample location and sample collection

**Fig. 1:**
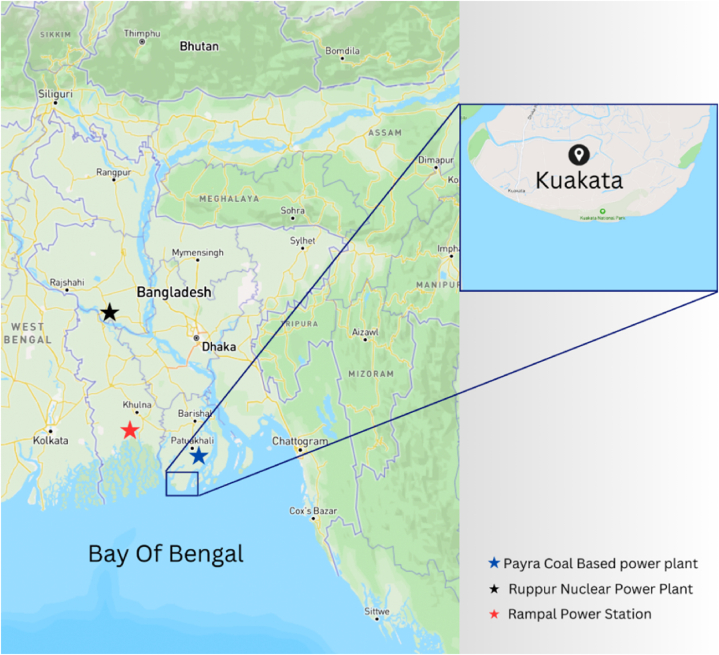
Geological location of Kuakata sea beach.

**Fig. 2:**
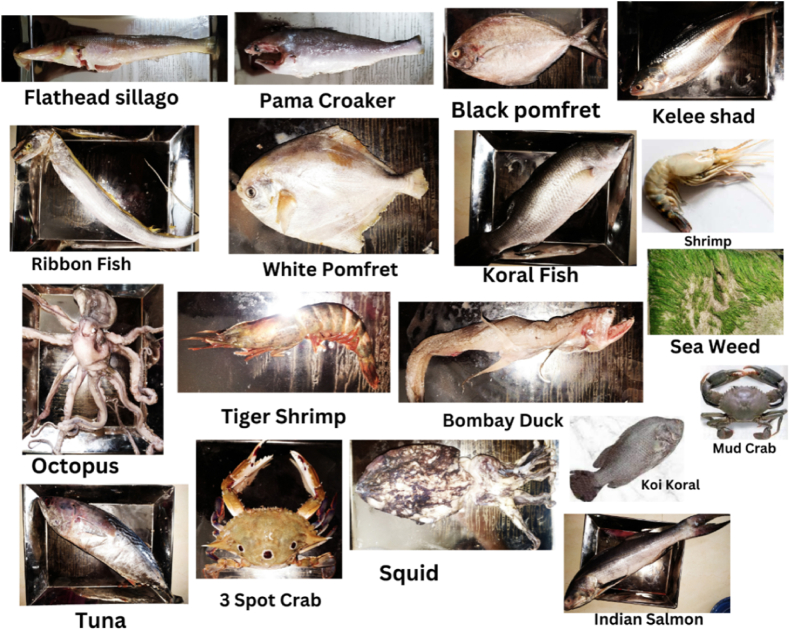
Fish, shellfish, mollusks and seaweed samples.

In order to measure the activity concentration of natural radionuclides and heavy metal contamination in marine species, eleven edible fish, four shellfish, and two mollusk species, a total of seventeen (n = 17) samples were collected from the local sea fish market in Kuakata, the northern part of the Bay of Bengal, Bangladesh, during the winter season in January 2023. Common seaweed was also collected from Kuakata's seashore. *Ulothrix flacca* is the most common and natural seaweed on the Kuakata coastline, even though there is no report of radioactivity or heavy metals in this seaweed. The geographical coordinates of Kuakata sea beach for latitude and longitude are 21.8031° N, 90.1823° E. The geographical position of the sampling area is shown in the following map (Fig. 1). The list of samples is shown in Table 1 and Fig. 2.

**Table 1:** Name of the fish, shellfish and mollusks samples collected from Kuakata.

Sl. No	Sample Code	Local Name	English Name	Scientific Name
1	FS-01	Bele fish	Flathead sillago	*Sillaginopsis panijus*
2	FS-02	Squid	Squid	*Loligo edulis*
3	FS-03	Octopus	Grey octopus	*Octopus rugosus*
4	FS-04	Samudrik chingri	Tiger shrimp	*Penaeus monodon*
5	FS-05	Poa	Pama croaker	*Otolithoides pama*
6	FS-06	Koi koral	Koi koral	*Acanthopagrus berda*
7	FS-07	Tin fota kankra	Three spot crab	*Portunus sanguinolentus*
8	FS-08	Chingri	Shrimp	*Penaeus semisulcatus*
9	FS-09	Loitta	Bombay-duck	*Harpadon nehereus*
10	FS-10	Hilsa shad	Kelee shad	*Hilsa kelee*
11	FS-11	Kada kankra	Mud crab	*Scylla serrata*
12	FS-12	Churi	Ribbon fish	*Lepturacanthus savala*
13	FS-13	Lakkha	Indian salmon	*Eleutheronema tetradactylum*
14	FS-14	Rup chada	Silver pomfret	*Pampus argenteus*
15	FS-15	Kala chada	Black pomfret	*Parastromateus niger*
16	FS-16	Tuna	Tuna	*Thunnus albacores*
17	FS-17	Koral	Sea bass	*Lates calcarifer*
18	SW-18	Seaweed	Seaweed	*Ulothrix flacca*

### 2.2 Sample preparation for gamma counting

The fish, shellfish and, mollusks samples were stored at a temperature of roughly −20 °C to ensure their preservation until they underwent additional laboratory processing. Before beginning any laboratory work, the fish samples were thoroughly cleaned three times with deionized water to remove any foreign contaminants. To assess the activity concentration in the edible portion of the samples, the fish flesh was sliced before being put in an appropriately sized stainless steel container lined with clear paper free of ash. Then, the viscera and shells were carefully removed. The samples were dried in a furnace where the temperature was gradually raised from 150 to 250 °C. After being dried, the samples were crushed, sieved to homogenize it, and stored in empty plastic cylinder containers that were clean, dry, and free of contamination. The seaweed sample was chopped up into tiny fragments and spread out on several pieces of brown paper to dry in the air. The samples were then dried in an electric oven set to 70 °C until they were friable. The samples were then ground into a powder using a grinder. To ensure the radioactive equilibrium between the uranium and thorium series and their short-lived progenies, the samples were securely kept in containers for a month [16].

### 2.3 Measurement of gamma activity

The HPGe Coaxial Detector was used in this investigation to detect radionuclides. The detector was placed in a cylindrical lead shielding device with a fixed bottom and sliding cover to reduce interference from ambient noise. With a 30 % relative efficiency, it was found that the energy resolution of the 1.33 MeV energy peak for ^60^Co was 1.67 keV at full-width half-maximum (FWHM). The energy calibration of the detector was carried out with standard point sources such as ^22^Na, ^57^Co, ^60^Co, ^88^Y, ^133^Ba, ^137^Cs, ^152^Eu, and so on. Efficiency data was also checked by a standard source which was made by combining ^152^Eu of known activity (Liquid form, 37000 Bq activity) with 1N HCl and manufactured in the same containers as the samples. Quality control (QC) of measurement was ensured with known activity concentrations of radionuclides from IAEA-TEL-2019 Proficiency Test.

A standardized counting time of 10,000 s was applied for both background and sample measurements to minimize uncertainty in net counts. The gamma spectra of each sample were analyzed, and unknown radionuclides were identified by comparing peak centroid energies with reference values from the literature. The activity of ^238^U was measured from the 609.06 keV and 1763.11 keV photo peaks of ^214^Bi. ^232^Th was estimated from the 910.77 keV, 2613.74 keV gamma peaks of ^228^Ac and ^208^Tl respectively. ^40^K was measured using the 1460.68 keV gamma peak from ^40^K itself. Identified radionuclides were quantified using the areas under the peaks, revealing the activity concentrations of ^238^U (^226^R), ^232^Th, and ^40^K in various samples. Specific activity, denoting the activity per unit mass of the sample, was thus defined.

The specific activity of individual radionuclides in fish, shellfish, mollusks and seaweed samples was calculated by the following equation (1),

(1) $$A=\frac{N}{I\times \varepsilon \times w}$$

Here, A is activity Bq/kg, N is net counts per second (cps) = Sample cps - Background cps, I is the gamma intensity, ε is counting efficiency, W is the sample mass in kg. Equation (2) was used to determine the minimal detectable activity concentration (MDAC) for the gamma-ray measuring system [17].

(2) $$\text{MDA}=\frac{K_{\alpha }\times \sqrt{B}}{\varepsilon \times p_{\gamma }\times T\times W}$$

where B, the background counts for the corresponding radionuclide, T is the counting period, p_γ_, the gamma ray transition probability, and W is the sample weight in kg. K_α_ is the statistical coverage factor, and its value is 1.64 (at the 95 % confidence level). The minimal detectable activity of ^238^U, ^232^Th, and ^40^K are found to be 0.65 Bq/kg, 0.57 Bq/kg, and 5.32 Bq/kg, respectively.

The total error, the sum of random and systematic errors, is termed uncertainty. In the context of activity measurement, the net count rate is the difference between the gross count rate (background count rate + sample count rate) and the background count rate. Every count rate has a standard deviation, and the net count rate's standard deviation may be given by using equation (3).

(3) $$\sigma =\pm \sqrt{\frac{A_{s}}{T_{s}}+\frac{A_{b}}{T_{b}}}$$

where σ is the standard deviation, $A_{s}$ is the ample count rate in cps, $A_{b}$ is the background count rate in cps, ${\;T}_{s}$ is sample counting time, ${\;T}_{b}$ is background counting time. The standard deviation of the ±1σ level was used to express the measurement errors.

### 2.4 Sample preparation for gross alpha and gross beta count

The studied samples were washed with deionized water three times. The samples were separated into edible muscle tissues which were utilized to measure the gross alpha and gross beta activity. To find the dry mass to wet mass ratio, the edible muscle tissues were weighed, oven dried at 150 °C to a consistent weight, powdered, and reweighed. Then, using a mixed solution of HNO_3_ and HCl (1:3), the powdered samples were wet-digested. H_2_O_2_ was added as needed to finish the digestion process. The samples were evaporated after digestion, and the residual precipitates were utilized in the following procedure [18]. For every measurement, a specific quantity of residue was taken from each sample and applied on the measuring trays' surface.

### 2.5 Gross alpha and gross beta activities calculation

A ZnS scintillation detector with a zinc sulfide layer attached to plastic, part of the MPC-2000-B-DP, was used to measure the gross alpha and gross beta activity. The following counting efficiency was used for activity calculation: Alpha efficiency: 36.8 %; beta efficiency: 41 %. Standard sources with defined activity, such as ^230^Th and ^90^Sr, were used to calibrate the detectors. Samples were monitored for gross alpha and beta activity over 120 min, and results were presented using standard deviation, activity, and count per minute. Data for both alpha and beta modes were collected, and the alpha and beta count rates were determined using a specified formula in equations (4) and (5).

(4) $$\text{Gross}\;\text{alpha}\;\text{activity},A\;\left(\text{DMP}\right)=\frac{A\;\left(\text{NET}\;\text{CPM}\right)\times 100}{A\left(\text{EFF}\right)}$$

where, A (DPM) = Net alpha disintegrations per min., A (NET CPM) = Net alpha counts per min., A (EFF) = Alpha efficiency percent.

(5) $$\text{Gross}\;\text{beta}\;\text{activity},B\;\left(\text{DMP}\right)=\frac{B\left(\text{NET}\;\text{DMP}\right)\times 100}{B\;\left(\text{EFF}\right)}$$

where, B (DPM) = Net beta disintegrations per min., B (NET CPM) = Net beta counts per min, B (EFF) = Beta efficiency percent.

### 2.6 Measurement of heavy metals

The chemicals utilized for sample digestion were of analytical grade. For heavy metals analysis, approximately 5.0 g of each sample, prepared on a dry basis, was weighed accurately and transferred into a 250 mL conical flask. Then 15 mL of 68 % HNO_3_ (Merck, Germany) and 65 % HClO_4_ (Merck, Germany) in 4:1 was added and heated on a hot plate in a fume hood until the solution turned colorless, indicating complete digestion. Digested samples were then allowed to cool, and 15 mL of distilled water was added. They were then filtered using an ashless Whatman filter into a 25 mL volumetric flask and made to mark with double distilled water. The fish, shellfish, mollusks and seaweed samples were analyzed for Fe, Zn, Mn, Pb, Cd, and Cr using Atomic Absorption Spectrophotometer (AAS) (iCE 3000 Series, Thermo Scientific, UK). Cold vapor atomic absorption spectrophotometer (Model Varian AA240 FS) was used to measure the mercury (Hg) content in the analyzed samples. The measurement of the analyzed heavy metals was based on the calibration curves. For every metal to be investigated, calibration curves were developed. The regression equations for each calibration curve were determined. In all cases, the regression coefficient (R^2^) was found between 0.995 and 0.999. The soundness of the analytical procedure was further reinforced by performing spike samples and obtaining recoveries. The analytical results were compared using replicate analysis. A reagent blank was analyzed after every five samples, and a standard solution was measured after every ten samples. The limit of detection (LOD) for the investigated heavy metals were 0.21 mg/kg (Fe), 0.13 mg/kg (Zn), 0.19 mg/kg (Mn), 0.21 mg/kg (Pb), 0.18 mg/kg (Cd), 0.21 mg/kg (Cr), and 0.08 μg/kg (Hg).

### 2.7 Radiation risk assessment method

The radiation risk can be evaluated by using different indices. In case of radiation risk in water, food/seafood samples annual effective dose/ingestion dose and excess lifetime cancer risk are used frequently. Generally, the ingestion of radionuclides depends on consumption rate of water or food/seafood and activity of radionuclides.

***Annual Effective Dose Rate (D):*** The measurement of activity concentration of radionuclide in food (Bqkg^−1^) and their multiplication by the masses of food consumed over a specific period (kgd^−1^ or kgy^−1^) yields the radiation doses ingested. The ingestion dose can then be estimated by applying a dose conversion factor (SvBq^−1^). The ingested dose is therefore administered by equation (6) [19].

(6) Annual effective dose (Svy^−1^) = Concentration (Bqkg^−1^) × Annual intake (kgy^−1^) × DCF (SvBq^−1^)

Standard dose conversion factor (DCF) is 0.2800 μSvBq^−1^ for ^238^U (^226^Ra), 0.2300 μSvBq^−1^ for ^232^Th, and 0.0062 μSvBq^−1^ for ^40^K in this case. Equation (7) can be used to get the total effective dose (Svy^−1^) by consumption [19].

(7) Total Annual Effective Dose = {C_R_ (^238^U) × I_F_ × E_D_} + {C_R_ (^232^Th) × I_F_ × E_D_} + {C_R_ (^40^K) × I_F_ × E_D_}

where IF is the annual intake (22.94 kgy^−1^) of radionuclide-containing fish and shellfish, ED is the ingestion dose conversion factor for radionuclides (SvBq^−1^), and CR is the concentration of radionuclides in ingested fish and shellfish (Bqkg^−1^).The intake rates for Bangladeshi consumers were taken from the “Year Book of Fisheries Statistics of Bangladesh [20] and the worldwide average annual effective dose which stands approximately at 2400 μSvy^−1^ [21,22].

***Excess Lifetime Cancer Risk (ELCR):*** The United States Environmental Protection Agency (USEPA) developed a system to attempt to evaluate the increased lifetime cancer risk associated with consumption of marine fish and crustaceans [23]. The increased lifetime cancer risk was computed using the following equation (8) [24].

(8) ELCR = A_ir_ × A_ls_ × R_c_

Here, ELCR, A_ir_, A_ls_, and R_c_ are the excess lifetime cancer risk, the annual intake of radionuclide (Bq), (According to Fisheries Resources Survey System, FRSS), Department of Fisheries, Ministry of Fisheries, the average daily consumption of fish and shellfish in Bangladesh is 22.94 kg/year 2018 [25] the average lifespan 72 yr (life expectancy of a male and a female are 70.6 years and 73.5 years, respectively in Bangladesh [20] and the mortality cancer risk coefficient (Bq^−1^), respectively. The values of mortality cancer risk coefficients are 9.56 × 10^−9^ Bq^−1^ for ^238^U, 2.45 × 10^−9^ Bq^−1^ for ^232^Th, and 5.89 × 10^−10^ Bq^−1^ for ^40^K. In general, the acceptable ELCR limit for ionizing radiation risk is 10^−3^ [23].

### 2.8 Health risk assessment of heavy metals

Various techniques for estimating health risks have been developed to assess the risk that consuming contaminated fish poses to humans. A common method for determining the quantity of pollutants ingested each day is the Estimated Daily Intake (EDI). The concentrations of potentially hazardous heavy metals in food and daily food consumption are directly correlated with the EDI, Target Hazard Quotient (THQ).

***Estimated Daily Intake (EDI):*** Estimated daily intake was measured by the following equation (9) in mgkg^−1^ body weight per day [26].

(9) $$\text{EDI}=\frac{E_{F}\times E_{D}\times F_{\text{IR}}\times C_{f}\times C_{M}}{W_{\text{AB}}\times AT_{n}}\times 10^{‐3}$$

where E_F_ is the frequency of exposure (365 days a year), E_D_ is the duration of exposure (72 years), F_IR_ is the ingestion rate (62.58 g per person per day) [25], C_f_ is the conversion factor (C_f_ = 0.208) to convert fresh weight to dry weight considering 79 % of the fish fillet's moisture content [26,27], C_M_ is the heavy metal concentration in fish fillet (mgkg^−1^ dry weight basis), W_AB_ is the average adult Bangladeshi person's body weight (60 kg) [28], and AT_n_ is the average exposure period for non-carcinogens [26].

***Target Hazard Quotient (THQ):*** THQ is a dimensionless, non-carcinogenic danger. The target hazard quotients (THQs) were used in this study to evaluate the non-carcinogenic health hazards related to eating fish and crustacean species. The THQs were calculated using the standard assumption for an integrated USEPA risk analysis, as follows equation (10) [23].

(10) $$\text{THQ}=\frac{E_{F}\times E_{D}\times F_{\text{IR}}\times C_{f}\times C_{M}}{W_{\text{AB}}\times AT_{n}\times \text{RfD}}\times 10^{-3}$$

E_F_, E_D_, F_IR_, C_f_, C_M_, W_AB_, and AT_n_ are explained in the earlier section. RfD is the reference dose of individual metal (mgkg^−1^day^−1^). RfD for Fe, Zn, Mn is respectively 0.7, 0.3, 0.14 mgkg^−1^day^−1^ [29]. The RfD is an estimate of the daily exposure to which the general population is susceptible throughout life without posing a serious risk of harmful consequences. The exposed population is unlikely to show overt negative consequences if the THQ is less than 1. There may be a health risk if the THQ is equal to or more than 1, in which case relevant actions and preventative measures are to be implemented [30].

***Hazard Index (HI):*** The total health risk (THQ) of each metal is added up, and this is called the hazard index (HI), which is used to quantify the total possible health risk associated with many metals. Exposure populations are unlikely to manifest overt negative effects if the THQ <1. The target hazard quotients (THQs) were calculated by using equation (11) [26].

(11) HI = THQMn + THQFe + THQZn + THQPb + THQCd + THQCr + THQHg

## 3 Results and discussion

The length, width and weight of fish, shellfish, mollusks and seaweed samples collected from the local sea fish market and seashore respectively in Kuakata, the northern part of the Bay of Bengal, Bangladesh are given in Table 2. The fishes are Flathead sillago, Pama croaker, Koi koral, Bombay-duck, Kelee shad, Ribbon fish, Indian salmon, Silver pomfret, Black pomfret, Tuna, Sea bass and the shellfish (crustaceans) are Tiger Shrimp, Three-spot Swimming Crab, Shrimp, Mud crab and the mollusks are Squid, Grey Octopus. The table shows a wide variety of physical properties (length) among fish, crustaceans and mollusks ranging from 5.33 cm to 55.88 cm in length, 6.00 g–1450.00 g in weight. The seaweed has a length of 3.81 cm and lowest in weight.

**Table 2:** Length, width and weight of collected samples.

Sample Code	English Name	Length (cm)	Width (cm)	Weight (g) per fish
FS-01	Flathead sillago	26.67	3.81	123.75
FS-02	Squid	17.78	20.32	232.20
FS-03	Grey octopus	33.02	10.41	335.00
FS-04	Tiger shrimp	5.33	0.76	6.20
FS-05	Pama croaker	20.32	5.08	111.11
FS-06	Koi koral	33.52	15.24	1235.00
FS-07	Three-spot crab	10.16	15.24	87.50
FS-08	Shrimp	13.20	2.79	45.00
FS-09	Bombay-duck	22.86	3.04	92.72
FS-10	Kelee shad	30.48	8.89	482.50
FS-11	Mud crab	14.73	17.78	101.30
FS-12	Ribbon fish	50.80	3.81	172.50
FS-13	Indian salmon	55.88	13.97	1450.00
FS-14	Silver pomfret	15.24	12.7	178.33
FS-15	Black pomfret	26.67	13.97	255.00
FS-16	Tuna	35.56	10.16	1020.00
FS-17	Sea bass	31.75	12.70	1060.00
SW-18	Seaweed	3.81	0.24	0.001

### 3.1 Gamma activity concentration

The gamma activity concentration of ^238^U, ^232^Th and ^40^K in collected fish, shellfish, mollusks and seaweed samples from the local sea fish market and seashore respectively in Kuakata, the northern part of the Bay of Bengal, Bangladesh were measured by HPGe detector, which are given in Table 3. The activity of ^226^Ra represents the activity of ^238^U because ^226^Ra is progeny of ^238^U decay series having secular equilibrium with parent.

**Table 3:** Gamma activity of^238^U,^232^Th and^40^K in Bq/kg in studied samples.

Sample Code	^238^U	^232^Th	^40^K
FS-01	20.9 ± 1.7	11.7 ± 0.9	196 ± 15
FS-02	15.2 ± 1.2	13.8 ± 1.1	141 ± 11
FS-03	17.2 ± 1.4	10.2 ± 0.8	152 ± 12
FS-04	19.9 ± 1.6	15.8 ± 1.2	233 ± 18
FS-05	18.4 ± 1.5	13.7 ± 1.0	167 ± 13
FS-06	13.7 ± 1.0	16.4 ± 1.3	177 ± 14
FS-07	15.9 ± 1.3	8.3 ± 0.6	183 ± 14
FS-08	22.0 ± 1.8	12.6 ± 1.0	143 ± 11
FS-09	18.4 ± 1.5	10.9 ± 0.8	164 ± 13
FS-10	19.8 ± 1.6	13.1 ± 1.0	174 ± 14
FS-11	25.3 ± 2.0	16.3 ± 1.3	223 ± 18
FS-12	18.6 ± 1.5	9.4 ± 0.7	176 ± 14
FS-13	24.6 ± 1.9	15.0 ± 1.2	235 ± 18
FS-14	23.5 ± 1.9	14.1 ± 1.2	231 ± 18
FS-15	21.4 ± 1.7	10.8 ± 0.8	243 ± 19
FS-16	19.8 ± 1.6	6.7 ± 0.5	181 ± 14
FS-17	17.8 ± 1.4	7.1 ± 0.5	166 ± 13
SW-18	22.8 ± 1.8	13.6 ± 1.0	202 ± 16
Average	19.7 ± 1.6	12.2 ± 0.9	188 ± 15

**Fig. 3:**
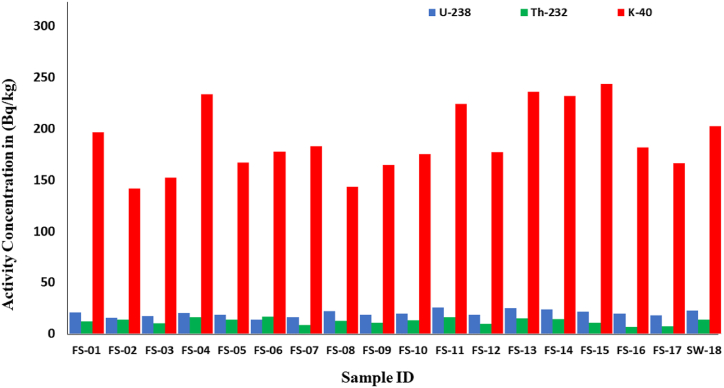
Gamma activity of ^238^U, ^232^Th and ^40^K in studied samples.

The bar graph (Fig. 3) shows a comparison of the activity concentration of three naturally occurring isotopes (^238^U, ^232^Th, and ^40^K) in eighteen different samples. The gamma activity concentration of ^238^U for analyzed fish, shellfish, and mollusk samples ranges from 25.3 ± 2.0 Bq/kg to 13.7 ± 1.0 Bq/kg, with an average value of 19.7 ± 1.5 Bq/kg. The activity concentration of ^232^Th ranges between 16.4 ± 1.3 Bq/kg to 6.7 ± 0.5 Bq/kg with an average value of 12.2 ± 0.9 Bq/kg, and the activity concentration of ^40^K ranges between 243 ± 19 Bq/kg to 141 ± 11 Bq/kg with an average value of 188 ± 15 Bq/kg. The highest values of ^238^U, ^232^Th, and ^40^K were found in samples FS-11, Mud crab (*Scylla serrata*), FS-06, Koi koral (*Acanthopagrus berda),* and FS-15, Black pomfret (*Parastromateus niger),* respectively. The seaweed has an activity of ^238^U, ^232^Th, and ^40^K of 22.8 ± 1.8 Bq/kg, 13.6 ± 1.0 Bq/kg, and 202 ± 16 Bq/kg, respectively. The average activity concentrations of ^238^U, ^232^Th, and ^40^K in analyzed samples were lower than the acceptable world average value (33 Bq/kg for ^238^U, 45 Bq/kg for ^232^Th, and 420 Bq/kg for ^40^K) [31].

**Fig. 4:**
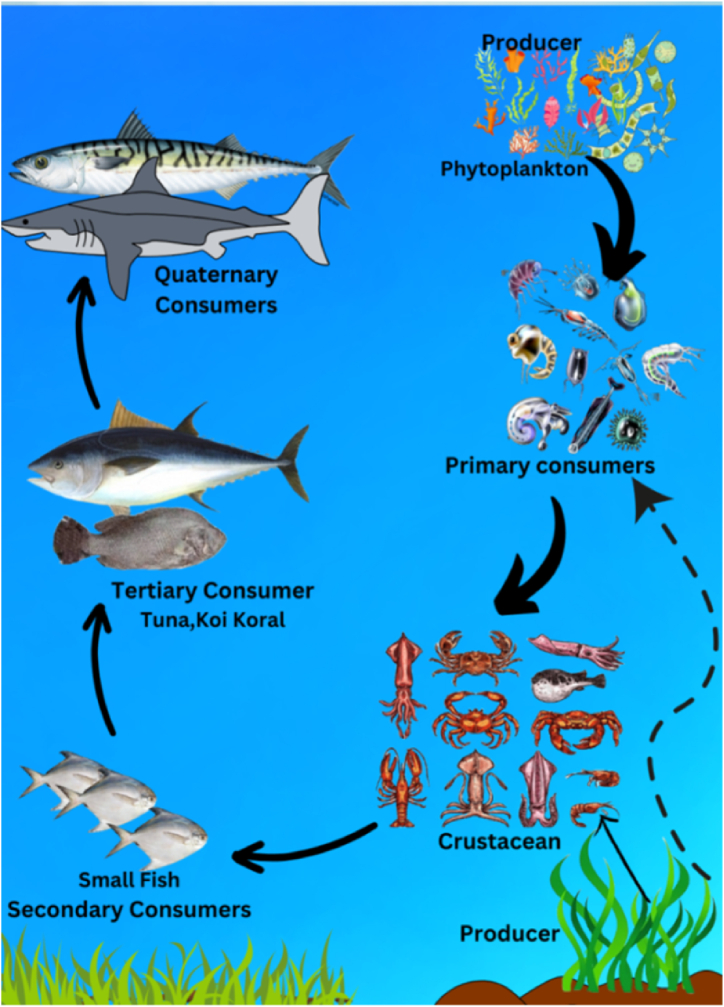
Food chain of marine organisms collected for analysis.

In comparison to ^232^Th and ^238^U, the majority of the fish samples have a greater gamma activity concentration of ^40^K. The reason for this is that because of its geochemical behavior, biological absorption, and dynamics of marine ecosystems, potassium-40 (^40^K) is more common in nature and the marine environment than uranium (^238^U) and thorium (^232^Th). Compared to ^238^U and ^232^Th, ^40^K is more easily carried by water, frequently absorbed by fish, and accumulates along food webs to produce larger concentrations. Because of the bioaccumulation of radionuclides into the both shell and flesh, the elevated gamma activity in crustaceans, Tiger shrimp (FS-04), three-spot swimming crab (FS-07), and mud crab (FS-11), compares to other samples. The sample with the highest concentration of ^238^U, together with considerable levels of ^232^Th and ^40^K, is the mud crab (*Scylla serrata*). Compared to small fish, gamma activity is significantly higher in secondary and tertiary fish. Large fish, such as Koi koral and Black pomfret, as well as small fish and crabs, typically consume fish eggs and larva [32]. As a result, this kind of fish has higher radioactivity than other fish. Fig. 4 illustrates the dietary habits of marine species and provides insight into how radionuclides migrate through the food chain.

Seaweed is an important bio-indicator in monitoring radioactive contamination in the marine environment. The activity concentration of naturally occurring radionuclides ^238^U, ^232^Th, and ^40^K in seaweed (*Ulothrix flacca*) sample collected from Kuakata Sea Beach was found to be 22.8 ± 1.8, 13.6 ± 1.0, and 202 ± 16 Bq/kg, respectively. That was the first approach to assessing the activity concentration of radionuclides in seaweed (*Ulothrix flacca*) at Kuakata Sea Beach, Bangladesh. The activity concentrations of ^238^U, ^232^Th, and ^40^K in the analyzed samples varied in the order of Shellfish > Seaweed > Fish > Mollusks, Seaweed > Shellfish > Mollusks > Fish, and Seaweed > Shellfish > Fish > Mollusks.

**Fig. 5:**
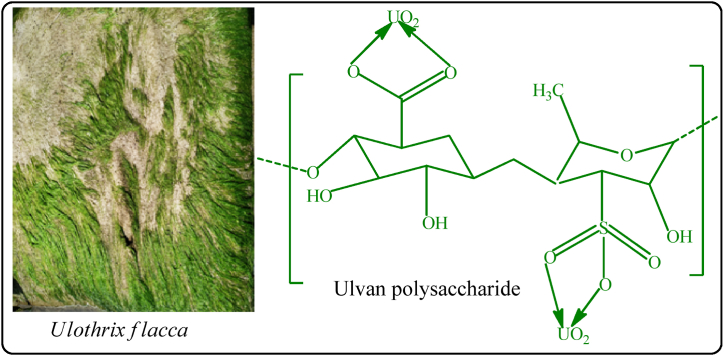
*Ulothirx flacca* and bioaccumulation of uranium in seaweed.

In all of the samples investigated, the activity concentration of ^238^U is higher than ^232^Th. This is because, as a result of its size and least variable oxidation state (IV), ^232^Th is less soluble in water than ^238^U [33]. Therefore, marine organisms are unable to accumulate more ^232^Th than ^238^U. The pH of oceanic seawater is somewhat higher than 8, and because of the dissolved organic acids and carbonate present, it has a buffering effect. Therefore, there are no conditions in the marine environment where U(VI) will precipitate. Thus, seawater acts as a sink for uranium [34]. Although uranium can exist in a variety of oxide forms, uranyl UO_2_^2+^, is by far the most prevalent species in marine environments. At seawater pH, uranyl will combine with carbonate to create complexes. [UO_2_(CO_3_)_3_]^4-^, [U(OH)_5_]^-^ are the main compound for pH ≥ 8 [35,36]. In the marine environment, the situation for thorium, Th(IV) is very different. The majority of thorium hydroxides, Th(OH)_4_ precipitate or adsorbed to solids found in the marine environment. This is also consistent with our measurements in marine species, where ^232^Th is found in lower amounts compared to ^238^U. The most important internal component that accounts for the remarkable ability to absorb inorganic matter from the environment is the distinct structure of the polysaccharides found in the cell walls of seaweed. Diverse seaweed families exhibit distinct mineral sorbent capacities due to the presence of varied structural polysaccharides, including fibrilar, nonfibrilar, and sulfated derivatives, which have varying numbers of binding sites for metal ions. Multifunctional polysaccharides provide robust ion-exchange characteristics [37]. According to research reports, Ulvan, tetradecanoic acid, 4-bromobenzoic acid, and other bioactive sulfated polysaccharides are present in the green algae *Ulothrix flacca* [38,39]. Consequently, ^238^U (VI) can be bioaccumulated into *Ulthrix flacca* more readily than ^232^Th (IV) because of its greater solubility and mobility in marine environment. This is achieved by interacting with binding sites of various organic acids and polysaccharides found in the green algae. A possible interaction of uranyl ion with ulvan polysaccharide is shown in Fig. 5.

### 3.2 Gross alpha and gross beta activity

The gross alpha and gross beta activity of the collected samples were measured using a Zinc sulphide scintillation detector and the measured values are presented in Table 4.

**Table 4:** Gross alpha and gross beta activity (Bq/kg) of the collected samples.

Sample Code	Gross alpha (Bq/kg)	Gross beta (Bq/kg)
FS-01	9.8 ± 1.5	27 ± 4
FS-02	7.0 ± 1.0	19 ± 3
FS-03	7.6 ± 1.1	21 ± 3
FS-04	11.6 ± 1.7	32 ± 5
FS-05	8.3 ± 1.2	23 ± 4
FS-06	8.8 ± 1.3	24 ± 4
FS-07	9.1 ± 1.4	25 ± 4
FS-08	7.1 ± 1.0	20 ± 3
FS-09	8.2 ± 1.2	23 ± 3
FS-10	8.7 ± 1.3	24 ± 4
FS-11	11.1 ± 1.6	31 ± 5
FS-12	8.8 ± 1.3	24 ± 4
FS-13	11.7 ± 1.7	33 ± 5
FS-14	11.5 ± 1.7	32 ± 5
FS-15	12.1 ± 1.8	34 ± 5
FS-16	9.0 ± 1.3	25 ± 4
FS-17	8.3 ± 1.2	23 ± 3
SW-18	10.1 ± 1.5	28 ± 4
Average	9.4 ± 1.4	26 ± 4

**Fig. 6:**
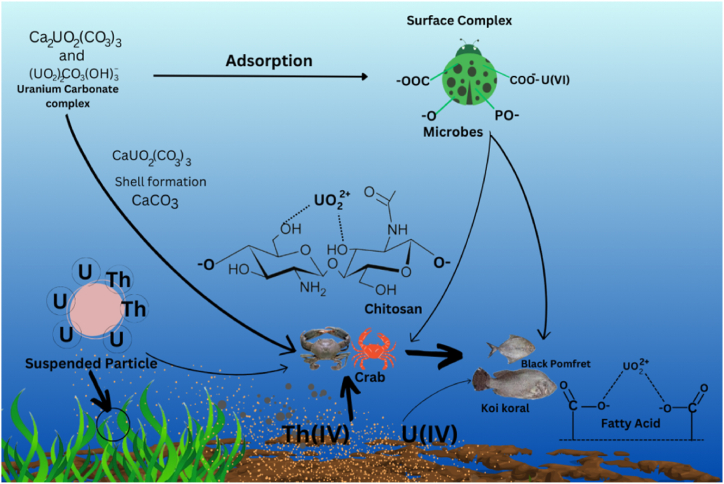
Theoretical model of bioaccumulation of radionuclides in marine organism.

The gross alpha activity of collected samples ranges from 12.1 ± 1.8 Bq/kg to 7.0 ± 1.0 Bq/kg with an average value of 9.4 ± 1.4 Bq/kg. The gross beta activity of the collected samples ranges from 34 ± 5 Bq/kg to 19 ± 3 Bq/kg, with an average value of 26 ± 4 Bq/kg. The higher gross beta activities in fish samples compared to gross alpha activities stem from a combination of factors, including the relative abundance of beta-emitting radionuclides, their longer decay characteristics, selective uptake and retention by fish, and contributions from non-radioactive sources. In marine environment, the primary sources of gross alpha activity are ^238^U and its progenies, ^210^Po, ^226^Ra, and occasionally the ^232^ Th day series; in contrast, the primary sources of gross beta activity are ^3^H, ^14^C, ^40^K, ^210^Pb, and ^228^Ra [[18], [40]]. The highest amount of gross alpha and gross beta activity was found in the FS-15 sample, Black pomfret (*Parastromateus niger*), and the lowest was found in the FS-02 sample, Squid (*Loligo edulis*), which is related to its carnivorous feeding type. FS-04, Tiger shrimp (*Penaeus monodon*), FS-07, Three spot crab (*Portunus sanguinolentus*), and FS-11, Mud crab (*Scylla serrata*) exhibited a noteworthy level of gross alpha and gross beta activity. These are omnivorous feeders that consume suspended organic particles, plankton, and algae. It has been suggested that radionuclide activity builds up and is largely caused by the plankton, its surroundings, and its bottom-feeding habit [[18], [41]]. It should be emphasized that a number of parameters, such as the size, age, and environment of the species, as well as eating patterns, rate of metabolism, water temperature, and depth, affect gross alpha and gross beta activity [18]. The current study area has a moderate degree of gross alpha and gross beta activity in the sample, according to the study mentioned above. Because alpha particles have the highest deposition power and the largest liner energy transfer, long-term exposure to them may be hazardous, even though the average gross alpha and gross beta activity in that area may not present any serious risks. Fig. 6 presents a theoretical model of radionuclide's bioaccumulation in marine species based on the dietary patterns of the currently analyzed samples and the radionuclide's geochemical behavior.

To obtain a more precise report that aids in our understanding of the level of activity concentration, the global radioactivity values of marine species can be compared. Table 5 presents a comparison of the activity concentration in this investigation with that in other studies.

**Table 5:** Comparative average activity of^238^U,^232^Th and^40^K and gross alpha, gross beta (Bq/kg) in the present study with worldwide other studies.

Location	^238^U	^232^Th	^40^K	Reference
Chattogram, Bangladesh	5 ± 2	67 ± 9	250 ± 50	[15]
Saint Martin Island, Bangladesh	10.86 ± 16.61	10.55 ± 13.5	364.92 ± 361.345	[13]
Karnaphuli River, Bangladesh	0.6109 ± 0.0001	0.0074 ± 0.000	24.9533 ± 0.0702	[14]
Tamilnadu, India	28.51 ± 17.77	239.58 ± 270.65	118.95 ± 56.24	[42]
Baghdad, Iraq	9.551	7.332	243.00	[43]
Yamaltu, Gombestate, Nigeria	6.70 ± 0.97	10.85 ± 1.74	30.19 ± 3.43	[44]
Niger State, Nigeria	37.22 ± 4.31	94.82 ± 3.82	384.98 ± 1.7	[45]
Jeddah, Saudi Arabia	1.63 ± 0.01	1.66 ± 0.02	208.13 ± 0.02	[46]
Kuakata	19.7 ± 1.5	12.2 ± 0.9	188 ± 15	This study
**Location**	**Gross alpha**	**Gross beta**		
Vietnam	35.6 ± 3.7	43.7 ± 2.0		[18]
Kuakata	9.4 ± 1.4	26 ± 4		This Study

### 3.3 Radiation risk assessment

Annual Effective Dose (AED) due to the consumption of ^238^U, ^232^Th and ^40^K through collected edible fish, shellfish and mollusks samples are presented in Table 6. The average annual effective dose of ^238^U, ^232^Th, and ^40^K due to consumption of seafood was calculated as 125 μSv/y, 64 μSv/y, and 26 μSv/y, respectively. FS-07 (Three spot Crab) has the lowest total annual effective dose rate, at 172 μSv/y, while FS-11 (Mud crab) has the highest total annual effective dose value, at 280 μSv/y. AED as a whole was found to be 216 μSv/y. The estimated AED value was significantly less than the world limit value of 2400 μSvy^−1^ [21]. The order of the total annual effective dose of studied samples was followed as, Shellfish > Fish > Mollusks. The average contributions of ^238^U, ^232^Th, and ^40^K to the total AED in studied seafood samples are 58 %, 29 %, and 12 %, respectively.

**Table 6:** Annual effective dose (AED) of different fish, shellfish and mollusks samples.

Sample Code	AED for^238^U μSvy^−1^	AED for^232^Th μSvy^−1^	AED for^40^K μSvy^−1^	Total AED μSvy^−1^
FS-01	134	62	27	224
FS-02	97	73	20	190
FS-03	110	54	21	186
FS-04	128	83	33	245
FS-05	118	72	23	214
FS-06	88	86	25	199
FS-07	102	43	26	172
FS-08	141	66	20	229
FS-09	118	57	23	199
FS-10	127	69	24	221
FS-11	162	86	31	280
FS-12	119	49	25	194
FS-13	158	79	33	271
FS-14	151	74	32	258
FS-15	137	57	34	229
FS-16	127	35	25	189
FS-17	114	37	23	176
Average	125	64	26	216

Excess Lifetime Cancer Risk (ELCR) due to the consumption of ^238^U, ^232^Th and ^40^K through collected edible fish, shellfish and mollusks samples is given in Table 7. The range of ELCR values for ^238^U is 0.305 × 10^−3^ to 0.165 × 10^−3^, for ^232^Th it is 0.197 × 10^−3^ to 0.081 × 10-3, and for ^40^K it is 0.180 × 10^−3^ to 0.105 × 10-3, respectively. The highest values of ELCR of ^238^U, ^232^Th, and ^40^K were found in samples FS-1 (Mud crab), FS-06 (Koi koral), and FS-15 (Black pomfret), respectively. The mean value of ELCR is 0.24 × 10^−3^ for ^238^U, 0.15 × 10^−3^ for ^232^Th, and 0.14 × 10^−3^ for ^40^K. The values of the ELCR of ^238^U are greater than those of ^232^Th and ^40^K, and these values are below the permissible threshold 1.00 × 10^−3^ [24]. The average contributions of ^238^U, ^232^Th, and ^40^K to the total ELCR in studied seafood samples are 47 %, 29 %, and 27.45 %, respectively. ^238^U is the most radiotoxic naturally occurring radionuclide and contributes the maximum amount of all ELCRs.

**Table 7:** Excess Lifetime Cancer Risk (ELCR) in studied samples.

Sample Code	ELCR of^40^K ( × 10^−3^)	ELCR of^238^U ( × 10^−3^)	ELCR of^232^Th ( × 10^−3^)	Total ELCR ( × 10^−3^)
FS-01	0.15	0.25	0.14	0.54
FS-02	0.10	0.18	0.17	0.45
FS-03	0.11	0.20	0.12	0.43
FS-04	0.17	0.24	0.19	0.60
FS-05	0.12	0.22	0.17	0.51
FS-06	0.13	0.17	0.19	0.49
FS-07	0.14	0.19	0.10	0.43
FS-08	0.11	0.27	0.15	0.53
FS-09	0.12	0.22	0.13	0.47
FS-10	0.13	0.24	0.16	0.53
FS-11	0.16	0.30	0.19	0.65
FS-12	0.13	0.22	0.11	0.46
FS-13	0.17	0.29	0.18	0.64
FS-14	0.17	0.28	0.17	0.62
FS-15	0.18	0.26	0.13	0.57
FS-16	0.13	0.24	0.08	0.45
FS-17	0.12	0.21	0.08	0.41
Average	0.14	0.24	0.15	0.51

### 3.4 Heavy metal concentration

The heavy metal concentration of the collected edible fish, shellfish, and mollusk species samples is presented in Table 8. The table lists the concentrations of heavy metals, specifically manganese (Mn), zinc (Zn), and iron (Fe), in different seafood samples with the labels FS-01 to FS-17. The concentrations of carcinogenic Pb (<0.21 mg/kg), Cd (<0.18 mg/kg), Cr (<0.21 mg/kg), and Hg (<0.08 μg/kg) were below the detection limits in all of the analyzed samples.

**Table 8:** Heavy metal concentration (mg/kg) in collected samples.

Sample ID	English Name	Heavy metal concentration (mg/kg)		
		Fe	Zn	Mn
FS-01	Flathead sillago	30.8 ± 1.3	39.8 ± 0.6	3.00 ± 0.25
FS-02	Squid	23.8 ± 1.2	38.4 ± 0.5	2.71 ± 0.28
FS-03	Grey octopus	35.7 ± 1.2	58.9 ± 0.6	3.08 ± 0.26
FS-04	Tiger shrimp	6.81 ± 1.24	19.7 ± 0.6	2.02 ± 0.25
FS-05	Pama croaker	27.2 ± 1.2	29.6 ± 0.5	2.95 ± 0.25
FS-06	Koi koral	146.8 ± 1.3	109.5 ± 0.5	10.8 ± 0.3
FS-07	Three spot crab	28.7 ± 1.2	111.0 ± 0.5	141.6 ± 0.3
FS-08	Shrimp	35.9 ± 1.2	47.7 ± 0.6	2.45 ± 0.25
FS-09	Bombay-duck	20.9 ± 1.2	38.5 ± 0.6	5.16 ± 0.26
FS-10	Kelee shad	53.6 ± 1.2	39.2 ± 0.5	9.6 ± 0.3
FS-11	Mud crab	104.0 ± 1.2	116.0 ± 0.6	162.3 ± 0.4
FS-12	Ribbon fish	16.8 ± 1.2	34.5 ± 0.6	2.12 ± 0.25
FS-13	Indian salmon	25.4 ± 1.3	23.9 ± 0.5	2.12 ± 0.27
FS-14	Silver pomfret	27.8 ± 1.2	36.6 ± 0.5	11.4 ± 0.3
FS-15	Black pomfret	18.0 ± 1.2	90.6 ± 0.6	9.6 ± 0.3
FS-16	Tuna	95.2 ± 1.3	35.5 ± 0.6	1.06 ± 0.25
FS-17	Sea bass	18.5 ± 1.2	20.5 ± 0.5	1.21 ± 0.21
Average	–	42.0 ± 1.3	52.3 ± 0.6	22.0 ± 0.3
FAO/WHO limit [47,48]	–	50	40	8

The average concentrations of Fe, Zn, and Mn were found to be 42.0 ± 1.3 mg/kg, 52.4 ± 0.6 mg/kg, and 22.0 ± 0.3 mg/kg, respectively. Koi Koral (FS-06) has the highest Fe and Zn concentrations ever measured at 146.8 ± 1.3 mg/kg and 109.5 ± 0.5 mg/kg, respectively, while Three spot crab (FS-07) has an exceptionally high Mn concentration of 141.6 ± 0.3 mg/kg. On the other hand, the Tiger shrimp (FS-04) has the lowest Fe (6.81 ± 1.24 mg/kg) and Zn (19.7 ± 0.6 mg/kg) concentrations. The Mn level (1.06 ± 0.25 mg/kg) is the lowest found in Tuna (FS-16). However, the average concentration of heavy metals followed the order of Zn > Fe > Mn in studied fish, shellfish and, mollusks samples. The recommended limit of Fe for human consumption, according to recommendation by FAO/WHO, is 50 mg/kg [47,48]. The permitted amount was exceeded by the fish species Koi koral (*Acanthopagrus berda*), Hilsa shad (*Hilsa kelee*), and Tuna (*Thunnus albacores*); the shellfish species Kada kankra (*Scylla serrata*) also exceeded this limit. The FAO/WHO has set a maximum allowed concentration of Zn in fish at 40 mg/kg [47,48]. However, in our study, It was observed that Zn concentrations in fish species including Koi koral (*Acanthopagrus berda*), Kala chada (*Parastromateus niger*), shellfish Tin Fota Kankra (*Portunus sanguinolentus*), Chingri (*Penaeus semisulcatus*), Kada kankra (*Scylla serrata*), and mollusks octopus (*Octopus rugosus*) exceeded the limit. Furthermore, the fish species Koi koral (*Acanthopagrus berda*), Kala chada (*Parastromateus niger*), Rup chada (*Pampus argenteus*), and shellfish Tin Fota Kankra (*Portunus sanguinolentus*), Chingri (*Penaeus semisulcatus*), and Kada kankra (*Scylla serrata*) exceeded the permissible limit for Mn, which was set by the FAO [47,48].

Mineral content in seaweeds is well known, and varies depending on the type of seaweed. The concentrations of Fe, Zn, and Mn in the seaweed (*Ulothrix flacca*) sample were found to be 15556 ± 10 mg/kg, 50.4 ± 2.0 mg/kg, and 555 ± 5 mg/kg, respectively. The Fe (1140–15030 mg/kg), Zn (10.60–33.46 mg/kg), and Mn (33.40–443.79 mg/kg) contents in red seaweed (*Hypnea* sp.), green seaweed (*Enteromorpha* sp.*),* and brown seaweed (*Sargassum* sp.*, Padina pavonica, Dictyota ciliolate,* etc.) collected from St. Martin's Island and Cox's Bazar of Bay of Bengal, Bangladesh, were studied by K. N. Chowdhury et al. [49]. The concentration of Fe, Zn, and Mn in our studied seaweed (*Ulothrix flacca*) sample is higher than that of the red seaweed, green seaweed, and brown seaweed. It has been noted that seaweeds function as specific biosorbents for various metals. It has been found that concentration factors (CFs) in aquatic algae are 10–20 times larger than those in terrestrial plants [37].

### 3.5 Health risk assessment of heavy metals

Table 9 presents a health risk assessment of heavy metals, specifically iron (Fe), zinc (Zn), and manganese (Mn), in various fish, shellfish, and mollusk species. The estimated daily intake (EDI) for each metal is well below the World Health Organization's limit value, indicating that individual metal consumption is not a significant concern. Additionally, the Hazard Quotient (HQ), which evaluates the risk of individual metals, is also below 1 for all fish samples, further supporting the conclusion that individual metal intake from these fish is within safe limits. Furthermore, for every fish, shellfish, and mollusks sample, the Hazard Index (HI), which totalizes the risk associated with all three metals, is consistently less than 1, suggesting that the intake of these metals in combination does not present a possible health concern. All of the study's samples, in summary, show values that are significantly lower than the Hazard Index (HI) and Threshold Hazard Quotient (THQ), indicating that eating these fish, shellfish, and mollusks does not pose a health risk due to heavy metal intake.

**Table 9:** Health risk assessment of heavy metals in collected samples.

Sample ID	EDI			THQ			HI
	Fe	Zn	Mn	Fe	Zn	Mn	
FS-01	6.6 × 10^−3^	8.6 × 10^−3^	7.0 × 10^−4^	9.4 × 10^−3^	2.8 × 10^−2^	4.7 × 10^−3^	4.2 × 10^−2^
FS-02	5.1 × 10^−3^	8.3 × 10^−3^	6.0 × 10^−4^	7.3 × 10^−4^	2.7 × 10^−2^	4.2 × 10^−3^	3.9 × 10^−2^
FS-03	7.7 × 10^−3^	1.2 × 10^−2^	7.0 × 10^−4^	1.1 × 10^−2^	4.2 × 10^−2^	4.8 × 10^−3^	5.8 × 10^−2^
FS-04	1.5 × 10^−3^	4.3 × 10^−3^	4.0 × 10^−4^	2.1 × 10^−3^	1.4 × 10^−2^	3.1 × 10^−3^	1.9 × 10^−2^
FS-05	5.9 × 10^−3^	6.4 × 10^−3^	6.0 × 10^−4^	8.4 × 10^−3^	2.1 × 10^−2^	4.6 × 10^−3^	3.4 × 10^−2^
FS-06	3.1 × 10^−2^	2.3 × 10^−2^	2.3 × 10^−3^	4.5 × 10^−3^	7.9 × 10^−2^	1.6 × 10^−2^	1.4 × 10^−2^
FS-07	6.2 × 10^−3^	2.4 × 10^−2^	3.0 × 10^−2^	8.9 × 10^−3^	8.0 × 10^−2^	2.1 × 10^−1^	3.0 × 10^−1^
FS-08	7.8 × 10^−3^	1.0 × 10^−2^	5.0 × 10^−3^	1.1 × 10^−2^	3.4 × 10^−2^	3.8 × 10^−3^	4.9 × 10^−2^
FS-09	4.5 × 10^−3^	8.4 × 10^−3^	1.1 × 10^−3^	6.5 × 10^−3^	2.7 × 10^−2^	8.0 × 10^−3^	4.2 × 10^−2^
FS-10	1.1 × 10^−2^	8.5 × 10^−3^	2.1 × 10^−3^	1.6 × 10^−2^	2.8 × 10^−2^	1.4 × 10^−2^	5.9 × 10^−2^
FS-11	2.2 × 10^−2^	2.5 × 10^−2^	3.5 × 10^−2^	3.2 × 10^−2^	8.3 × 10^−2^	2.5 × 10^−1^	3.6 × 10^−1^
FS-12	3.7 × 10^−3^	7.5 × 10^−3^	5.0 × 10^−4^	5.2 × 10^−3^	2.4 × 10^−2^	3.3 × 10^−3^	3.3 × 10^−2^
FS-13	5.5 × 10^−3^	5.2 × 10^−3^	5.0 × 10^−4^	7.9 × 10^−3^	1.7 × 10^−2^	3.3 × 10^−3^	2.8 × 10^−2^
FS-14	6.0 × 10^−3^	7.9 × 10^−3^	2.5 × 10^−3^	8.6 × 10^−3^	2.6 × 10^−2^	1.7 × 10^−2^	5.2 × 10^−2^
FS-15	3.9 × 10^−3^	1.9 × 10^−2^	2.1 × 10^−3^	5.6 × 10^−3^	6.5 × 10^−2^	1.4 × 10^−2^	8.6 × 10^−2^
FS-16	2.0 × 10^−2^	7.7 × 10^−3^	2.0 × 10^−4^	2.9 × 10^−2^	2.5 × 10^−2^	1.7 × 10^−3^	5.6 × 10^−2^
FS-17	4.0 × 10^−3^	4.4 × 10^−3^	3.0 × 10^−4^	5.7 × 10^−3^	1.4 × 10^−2^	1.9 × 10^−3^	2.2 × 10^−2^
Average	9.1 × 10^−3^	1.1 × 10^−2^	4.8 × 10^−3^	1.3 × 10^−2^	3.7 × 10^−4^	3.4 × 10^−2^	8.4 × 10^−2^
TDI^a^ [50]	0.8	0.3	0.14	<1	<1	<1	<1

a Tolerable Daily Intake.

## 4 Conclusion

The radioactivity of natural occurring radionuclides and the concentration of heavy metals were investigated in various economically important edible fish, shellfish, mollusks, and a common seaweed from the northern region of the Bay of Bengal, Kuakata, Bangladesh. The activity concentrations of ^238^U, ^232^Th, and ^40^K were found to be 19.7 ± 1.5 Bq/kg, 12.2 ± 0.9 Bq/kg, and 188.6 ± 15.0 Bq/kg, which fall within world average values, with the higher ^40^K value attributed to natural abundance. The predominance of ^238^U over ^232^Th suggests the specific bioaccumulation of uranium into the studied samples and particular geological features in the studied area. By comparing the current results with published data from various parts of the world, including comparable research conducted in the Bay of Bengal, it can be inferred that the analyzed samples contain a moderate level of radioactive elements. However, the current study shows that there is still not much threat to the public's health from the annually effective dosage and lifetime cancer risk associated with consuming fish, shellfish, and mollusks from the Bay of Bengal. Fish, shellfish, and mollusks under study all remain safe for consumption by humans even though some of the samples have heavy metal concentrations of Fe, Zn, and Mn over FAO/WHO recommended permissible levels. Additionally, the concentration of carcinogenic Pb, Cd, Cr, and Hg were below detection limits. The geochemical nature of the radionuclides and the feeding behaviors of marine organisms were used to explain the possible mechanisms of radionuclide and heavy metal bioaccumulation in seaweed and other marine species under study. Moreover, this study provides crucial baseline data for future regulatory considerations and monitoring efforts of marine pollution, particularly in light of the operation of the Rooppur Nuclear Power Plant and the proposed establishment of another nuclear power plant in the coastal region of Bangladesh. In the future, similar studies with numerous seafood and seaweed samples and in different seasons should be planned and conducted to track any changes in the Bay of Bengal's marine pollution.

## Funding

Funded by University Grant Commission (10.13039/100015747UGC), Bangladesh.

## Data availability

The data presented in this study are contained within the article.

## CRediT authorship contribution statement

**Samin Yeasar Risal:** Writing – original draft, Investigation, Formal analysis, Data curation. **Saiful Islam:** Writing – original draft, Supervision, Funding acquisition, Formal analysis, Conceptualization. **Jannatul Ferdous:** Methodology, Investigation, Formal analysis. **Md Nure Alam Siddik:** Investigation, Formal analysis. **Pradip K. Bakshi:** Writing – original draft, Supervision, Funding acquisition.

## Declaration of competing interest

The authors declare that none of the work reported in this study could have been influenced by any known competing financial interests or personal relationships. During the preparation of the manuscript we did not use any AI –assisted technologies.
